# Neurosteroids: mechanistic considerations and clinical prospects

**DOI:** 10.1038/s41386-023-01626-z

**Published:** 2023-06-27

**Authors:** Jamie L. Maguire, Steven Mennerick

**Affiliations:** 1 Department of Neuroscience, Tufts University School of Medicine, 136 Harrison Ave, Boston, MA 02111 USA; 2 Department of Psychiatry and Taylor Family Institute for Innovative Psychiatric Research, Washington University in St. Louis School of Medicine, 660 S. Euclid Ave., St. Louis, MO 63110 USA

**Keywords:** Ion channels in the nervous system, Depression

## Abstract

Like other classes of treatments described in this issue’s section, neuroactive steroids have been studied for decades but have risen as a new class of rapid-acting, durable antidepressants with a distinct mechanism of action from previous antidepressant treatments and from other compounds covered in this issue. Neuroactive steroids are natural derivatives of progesterone but are proving effective as exogenous treatments. The best understood mechanism is that of positive allosteric modulation of GABA_A_ receptors, where subunit selectivity may promote their profile of action. Mechanistically, there is some reason to think that neuroactive steroids may separate themselves from liabilities of other GABA modulators, although research is ongoing. It is also possible that intracellular targets, including inflammatory pathways, may be relevant to beneficial actions. Strengths and opportunities for further development include exploiting non-GABAergic targets, structural analogs, enzymatic production of natural steroids, precursor loading, and novel formulations. The molecular mechanisms of behavioral effects are not fully understood, but study of brain network states involved in emotional processing demonstrate a robust influence on affective states not evident with at least some other GABAergic drugs including benzodiazepines. Ongoing studies with neuroactive steroids will further elucidate the brain and behavioral effects of these compounds as well as likely underpinnings of disease.

## Introduction

The recent development of rapid-acting antidepressants has changed the landscape for the treatment of depression. Rapid antidepressant actions offer the opportunity to re-envision treatment strategies for depression, enabling an episodic treatment approach rather than a prophylactic approach which would revolutionize treatment options for patients.

These newly developed, rapidly acting antidepressants have novel mechanisms of action which differ from the traditional antidepressant classes, such as SPRAVATO® which acts on NMDA receptors and ZULRESSO® (brexanolone) which is a neuroactive steroid (NAS) and synthetic formulation of allopregnanolone which acts as a positive allosteric modulator (PAM) at GABA_A_ receptors (GABA_A_Rs). The antidepressant effects of these compounds offer new avenues for further treatment development targeting these novel targets as well as offer new insights into the pathophysiological mechanisms of disease.

To set the stage for discussion of risks and opportunities and mechanism of action, we briefly review the clinical data that resulted in recent approval of brexanolone for treatment of women with postpartum depression, and we give a status update of trials testing efficacy in men and women with depression. Brexanolone, an intravenous, synthetic formulation of allopregnanolone demonstrated antidepressant effects in women with severe postpartum depression (Hamilton Rating Scale for Depression [HAM-D] total score ≥26) in a double-blind, randomized, placebo-controlled trial across four different sites, demonstrating a significant reduction in HAM-D total score from baseline, albeit in a small number of patients (21: 10 brexanolone, 11 placebo) [1]. The antidepressant effects of brexanolone in women with postpartum depression was repeated in two multicenter, double-blind, randomized, placebo-controlled, phase 3 trials, both studies demonstrating a significant reduction in total HAM-D scores [2]. Brexanolone treatment was also demonstrated to achieve a more rapid and effective antidepressant effect as well as reduce anxiety and insomnia in patients [3]. It is interesting to note not only the rapid antidepressant effects of these compounds, but also the durability of the antidepressant effects, lasting at least 30 days [1–3]. On March 19, 2019, ZULRESSO® (brexanolone) was approved by the U.S. Food and Drug Administration (FDA) for the treatment of postpartum depression.

Based on the robust antidepressant effects of brexanolone, a similar, but orally available compound, zuranolone was developed and the antidepressant potential was evaluated in patients with postpartum depression or major depressive disorder. Similar to brexanolone, a 2-week treatment with zuranolone demonstrated a significant reduction in HAM-D score and Hamilton Rating Scale for Anxiety score in women with postpartum depression in a phase 3, double-blind, randomized, placebo-controlled clinical trial [4]. A follow-up phase 3, double-blind, randomized, placebo-controlled trial with a once-daily oral zuranolone treatment for 14 days increased concurrent remission of depressive and anxiety symptoms at days 3, 15, and 45 [5]. Zuranolone was shown to exert antidepressant effects in a double-blind, phase 2 trial in patients with major depressive disorder [6]. Improved quality of life was reported in patients with major depressive disorder in a randomized, placebo-controlled phase 2 trial [7]. Similar to brexanolone, zuranolone also exerted rapid and sustained antidepressant effects, lasting up to 45 days [4, 5]. On December 6, 2022, Biogen Inc. and Sage Therapeutics announced the submission of a New Drug Application (NDA) to the FDA for the use of zuranolone for the treatment of major depressive disorder and postpartum depression. This application was based on data from the LANDSCAPE program for major depressive disorder treatment, which includes five studies (MDD-201B, MOUNTAIN, SHORELINE, WATERFALL, and CORAL), and the NEST program for postpartum depression which included two studies (ROBIN and SKYLARK). At the time of this publication, the status of this application is unknown.

These are breakthrough studies which transform our current thinking regarding antidepressant treatment. It is critically important to understand how these compounds exert their rapid and persistent antidepressant action not only for the development of future treatments but also to obtain a better understanding of the underlying neurobiology of disease. In this section we will fully consider the advantages, disadvantages, and potential concerns regarding these rapid-acting antidepressants, discuss the unique features of GABA_A_R PAMs, possible non-GABAergic impacts of NAS, and potential role of network dynamics in the therapeutic properties of these compounds. Collectively, this contribution highlights potential novel mechanisms of action of these compounds which will inform further therapeutic development.

## Neuroactive steroids: strengths, weaknesses, opportunities and threats to clinical use

### Strengths

NAS are naturally-occurring compounds representing an attractive treatment strategy. NAS were approved in 2019 for treatment of postpartum depression based on a steroid replacement hypothesis [8]. Indeed, there is interest in this approach for treating other neuroendocrine disorders in women [9], and neurosteroid deficiency may characterize a subset of individuals with major depressive disorder in both sexes as described further below. At the same time, there has been an interest in exploiting glutamate and GABA transmitter systems in depressive disorders to circumvent limitations of SSRIs and other first-line treatments for depressive and mood disorders [10]. Given the especially highly potent and efficacious effects of NAS at GABA_A_Rs, the field is interested in expanding usage of NAS for disorders beyond neuroendocrine disorders. By targeting a mechanism distinct from conventional treatments, NAS, like ketamine and other novel approaches, might benefit patients who are not helped by traditional treatments (up to a third of patients). We are fortunate to have witnessed this turnaround given that only a decade ago, many warnings were given about the retreat of the pharmaceutical industry from neuropsychiatric indications [11–13].

Additional strengths of the NAS approach include rapidity of action. If indeed GABA_A_R PAM effects are important for behavioral effects, NAS readily pass the blood-brain barrier to have direct and immediate impact on signaling. By directly binding sites on heteropentameric GABA_A_Rs, NAS will immediately influence signaling, with no delay for second messenger signaling, transcription, and other slow processes. Despite the steroid structure, allopregnanolone and related NAS appear to have little to no effect on classical intracellular steroid receptors [14, 15]. Compared with benzodiazepines, NAS may target receptors of different subunit combinations (see below), thereby impacting different cell classes and different networks than benzodiazepines.

As noted above, a single administration of NAS has persisting, durable effects on nervous system function, well beyond the presence of drug in the brain. Benefit in women treated with 60 h infusion lasted up to 30 days (see above). Therefore, another strength of NAS is this longevity of action. The combination of rapidity and persistence has obvious advantageous clinical ramifications. However, it raises mechanistic questions. Do GABA_A_R effects trigger longer lasting changes to nervous system function that are responsible for maintaining the therapeutic benefit, or is the trigger related to a different target? After all, GABA_A_Rs are not especially well known for triggering plasticity. Similarly, do the effects responsible for maintaining the therapeutic benefit after drug has dissipated involve GABA_A_Rs, or are these effects independent of GABA_A_R signaling? These are important questions to address for optimizing effects of NAS: limiting side effects and maximizing benefit.

### Weaknesses

A major weakness is alluded to immediately above. Without a detailed understanding of mechanism of action, it is difficult to optimize analogs to limit off-target effects and screen for effectiveness using mechanism-based assays. This gap also reveals limits in our understanding of disease mechanisms. This weakness is of course not unique to neurosteroids but plagues virtually all psychotropic drugs.

To date, brexanolone is approved for postpartum depression, but its utility for other psychiatric indications, including major depressive disorder, is unclear, as outlined above. Trials for seizures, insomnia and essential tremor are also underway [16].

Another weakness of NAS is that the approved compound brexanolone/ZULRESSO® utilizes a Captisol formulation and 60 h intravenous infusion. Thus, it is limited to inpatient treatment. Development of the oral compound Zuranolone, described above would mitigate this weakness [4, 17, 18]. However, the maintenance of therapeutic effect of Zuranolone has emerged as a contentious issue [19].

### Opportunities

NAS in clinical development are similar to (Zuranolone) or identical to (brexanolone/allopregnanolone) endogenous neurosteroids. One opportunity for NAS that could circumvent formulation challenges is the targeting of enzymatic steps from cholesterol to allopregnanolone production. In principle, drugs or genetic therapies could be developed to alter neurosteroidogenesis, allowing the periphery and/or brain to produce its own drug. Another opportunity that exploits endogenous synthetic machinery is precursor loading. Systemic administration of the progesterone results in rapid anesthesia, attributable to progesterone’s conversion to allopregnanolone [20]. Precursors themselves could have beneficial effects [21] and thus could synergize with metabolites to maximize therapeutic impact.

Another opportunity involves the large catalog of synthetic NAS with GABA_A_R actions. The Covey group has designed and synthesized hundreds of analogs over the last few decades, and other groups are interested as well [22, 23]. An effective screening regimen could unearth compounds within this group that may have superior properties to the compounds tested to date, especially given the potential tunability of effects at GABA_A_Rs [24], a leading candidate to mediate benefit.

The present review focuses on mood disorders, including postpartum depression and major depressive disorder, since clinical trials are most advanced for these disorders. However, we note that GABA_A_R inhibition has been implicated in several other neuropsychiatric disorders (e.g., schizophrenia, epilepsy, intellectual and developmental disorders, substance use disorders). The neuroprotective properties of neurosteroids described below also suggest potential use in neurodegenerative disorders, such as Alzheimer’s disease, stroke, and traumatic brain injury. Compounds with similar GABA_A_ receptor subunit selectivity to NAS have also been of interest for sleep [16]. Fittingly, there is interest in NAS and clinical trials are underway for many of these indications [16, 25–27] (see also ClinicalTrials.gov).

### Threats

Targeting a transmitter system as widespread as the GABA system has inherent risk. By titrating dosage and fostering subunit selectivity or other unique mechanisms (described further below), detrimental effects such as sedation and abuse liability, which plague other GABA_A_R PAMs such as barbiturates and benzodiazepines, may be avoided. However, this magic shotgun approach [28] is not fully proven for NAS. In fact, allopregnanolone and pregnanolone in preclinical studies have shown reinforcing properties and indications of abuse liability, with tolerance related to the dosage and likely dosing frequency [29].

Use of brexanolone in women with postpartum depression increased dizziness and somnolence over the placebo arm and 4% of patients developed loss of consciousness. These side effects are worrisome in that they echo effects of benzodiazepines, with attendant abuse liability and other drawbacks. On the other hand, many patients in the clinical trial were co-medicated with other psychotropics, which could have contributed to the side effect. Caution has been encouraged in interpreting the “antidepressant” effects of brexanolone. Without additional study, antidepressant benefit is readily confused with reinforcing drug properties or effects on sleep (which can have direct impact on mood), a distinction that the clinical trials were not designed to make [30]. Dependence and withdrawal also remain understudied. Thus, while there is reason to think that GABA_A_R effects for benzodiazepines and NAS will differ based on the sub-classes of receptors targeted, liabilities of NAS remain unclear.

## Comparison of NAS with other GABA_A_R PAMs

Allopregnanolone and other NAS may achieve mood altering effects through their action as GABA_A_R PAMs [22, 31–33]. To contextualize allopregnanolone’s difference from other GABA_A_R PAMs, we first briefly review GABA_A_R composition, anatomical localization, physiological/biophysical properties, and pharmacological selectivity. We focus on two populations of receptors, defined by receptor subunit differences that may be relevant to allopregnanolone selectivity.

GABA_A_Rs are heteropentameric chloride channels that are activated by the neurotransmitter GABA and are resident on the plasma membrane of virtually all neurons in the CNS. They play an essential role in maintaining circuit function [34–37]. In general, the opening of the chloride channel by GABA binding promotes inhibition through chloride influx through the channel. Dysfunction of GABA_A_Rs has been implicated in the pathogenesis of neurological and neuropsychiatric diseases including epilepsy, autism, and depression [38–40]. Nineteen GABA_A_R subunit genes exist (α1-6, β1-3, γ1-3, δ, ε, ρ1-3, π, and θ); however, most native receptors are composed of two α, two β, and a variable fifth subunit that is not required for channel activation [41, 42]. This fifth subunit is most typically a γ (usually γ2) or δ subunit. Subunit combination, including α and β subvariants but especially the fifth γ2 or δ subunit, strongly influences anatomical, physiological and pharmacologic properties of the receptor.

γ2-containing receptors are expressed in many cell types, but δ expression is restricted to relatively few cell classes, including pivotal circuit relays such as dentate granule neurons of the hippocampal formation, cerebellar granule neurons, and thalamocortical neurons. The subunit is also enriched in parvalbumin-expressing interneurons and some pyramidal cell classes of the neocortex [43]. δ subunits preferentially assemble with α4 or α6 subunits in cell types that express these α subunits [44, 45]. By contrast, in δ-expressing interneurons, the subunit appears to pair with the more ubiquitous α1 subunit since neither α4 nor α6 is expressed in this cell class [46–49].

Regarding sub-cellular localization, GABA_A_Rs containing a γ2 subunit dominate occupation of synaptic territory opposite GABAergic presynaptic terminals. The trafficking of γ2-containing receptors includes interaction of γ2 with anchoring proteins at the synapse [50–52]. By contrast, δ-containing receptors are excluded from synapses and occupy perisynaptic and extrasynaptic locations [53].

These differences in sub-cellular location (synaptic vs. perisynaptic) also appear to partly drive differences in function. Receptors containing γ2 tend to activate and decay more quickly than receptors containing δ subunits, reflecting in part different distances from release sites but also biophysical differences [54–56]. δ-Containing receptors typically exhibit responsivity to lower GABA concentrations than γ2-containing receptors (i.e., they have a lower EC_50_ for GABA) but blunted maximum responses to GABA (lower efficacy) [54, 57]. This lower efficacy may be important to the pharmacological effects of NAS and other PAMs, described further below.

The difference in location and higher sensitivity to GABA give δ-containing receptors a preferential role in generating a low-amplitude, tonic inhibition driven by low concentrations of ambient GABA. Despite the small amplitude of tonic current, tonic inhibition can robustly influence neuronal excitability [58–60]. By contrast, phasic (synaptic) inhibition is mainly generated by γ2-containing receptors at synapses.

Allopregnanolone can modulate both tonic and phasic inhibition through its ability to potentiate many different populations of GABA_A_Rs, but it may have preferential effects based on the properties of extrasynaptic δ-containing receptors [61, 62]. Here we compare allopregnanolone actions, including subunit selectivity, with actions of barbiturates and classical benzodiazepines, which perhaps represent more familiar classes of GABA_A_R PAMs.

Much evidence suggests that allopregnanolone may selectively modulate δ-containing receptors, thereby primarily enhancing tonic current at low concentrations presumably relevant for effects on mood [59, 61, 63]. This would make NAS effects distinct from benzodiazepines, which require a γ subunit [64, 65]. First, mice deficient in δ subunits show loss of hypnotic sensitivity (sleep time) to NAS but not to other GABA_A_R PAMs [66]. Second, deletion of the δ subunit reduces tonic current in many relevant neuronal classes [59].

On the other hand, some evidence from concatemeric receptors and pharmacoresistant receptors in mice suggests that δ-containing receptors do not preferentially underlie the actions of allopregnanolone [62, 67]. Furthermore, the preferential effect of NAS results from the low efficacy of GABA acting at δ containing receptors [68], and other PAMs including propofol, etomidate, and pentobarbital have this same characteristic [54, 69–71]. In summary, effects of allopregnanolone on GABA_A_Rs are distinct from benzodiazepines, but the distinction from other GABA-active anesthetics remains unclear.

**Fig. 1:**
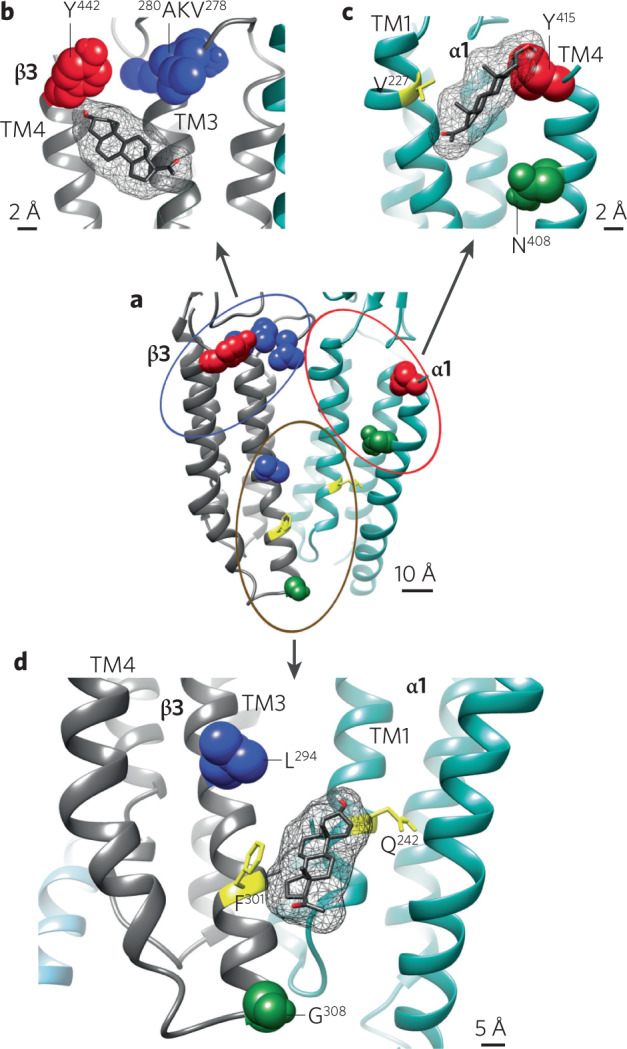
Sites of neurosteroid interaction with GABA_A_ receptors. Allopregnanolone (mesh structure) in the three neurosteroid binding sites (**b**–**d**) identified by photolabeling with photolabile NAS analogs. **a** (center): the six sites identified by photolabeling are grouped into three clusters: inter-subunit sites β3(+)/α1(−) (brown circle, detailed in **d**), β3 intra-subunit sites (blue circle, detailed in **b**), and α1 intra-subunit sites (red circle, detailed in **c**). Residues photolabeled by different analogs are colored red, green, and blue respectively. This figure is reproduced from [75].

If GABA_A_R PAM activity is important for clinical benefit, another approach to developing evermore improved NAS compounds to understand the binding sites on GABA_A_R for this class of compound. NAS appear to access transmembrane sites on the GABA_A_R multimer [72–74], with several functional intra-subunit and inter-subunit sites identified [75] (Fig. 1). Because the functional impact of binding each site differs [24], there is promise for a rich pharmacology that exploits differential access to the sites to customize pharmacodynamic effects. However, from the perspective of the receptor, relative affinities of the multiple sites for neurosteroids are not yet clear. However, mutagenesis studies have shown that a single neurosteroid site may suffice for full potentiation [76]. As broad spectrum potentiators, neurosteroids show relatively little difference in affinity for receptors of different subunit composition. As described elsewhere, differences attributed to the incorporation of the δ subunit appear mainly related to the low efficacy of GABA; NAS mainly increase agonist efficacy [54, 68].

From the ligand perspective, physiological pharmacology experiments have revealed important components of the pharmacophore that are important for full activity [31, 77–79]. These include a 3α hydrogen bond donor and a hydrogen bond acceptor at carbon 11 of the steroid. Interestingly, activity at GABA_A_ receptors is not markedly altered by stereochemistry of the reduction at carbon 5. Potency and to some degree efficacy of steroid effects can be manipulated by alterations to many other regions of the steroid molecule.

## Possible non-GABA_A_R targets of relevance

**Fig. 2:**
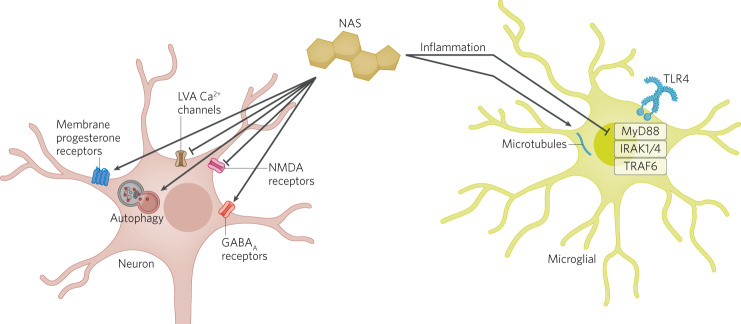
Schematic of non-GABA actions of NAS (yellow steroid structure). Depicted is a neuron (left) and microglial cell (right). Some targets may be found in multiple cell classes, so the relevant cell type is not known in all cases. See text for details.

There is reason to think that NAS benefit may arise at least in part through non-GABAergic mechanisms. NAS have ameliorative behavioral effects in female mice lacking δ subunits [80]. Further, other GABA_A_R PAMs described above, including those with very similar GABA_A_R sub-selectivity, are not known to have the same clinical benefit as NAS. In direct studies, some NAS appear to engage other targets, as described below. Therefore, non-GABA_A_R targets should be considered when evaluating mechanistic underpinnings of benefit. We briefly review other ion channel targets, intracellular kinases, and protein expression mechanisms, with particular focus on anti-inflammatory mechanisms (Fig. 2).

Some NAS classes negatively modulate NMDARs [81–85], which could impart clinical benefit akin to the NMDAR antagonist ketamine. A few compounds possess GABA_A_R PAM effects in combination with NMDAR negative modulation [84, 85]. It is tempting to consider that this constellation of effects might yield an advantageous brexanolone-like plus ketamine-like double hit. However, to date pharmacokinetic properties of such compounds have proven limiting.

Other work demonstrated that some NAS influence low-voltage activated (T-type) calcium channels. Allopregnanolone is an LVA inhibitor at sufficiently high concentrations [86, 87]. Medicinal chemistry efforts have resulted in NAS selectivity for LVA calcium channels over GABA_A_Rs [88]. It remains unclear how combined effects at LVA channels and GABA_A_Rs affect behaviors relevant to mood.

NAS can also exert indirect effects on numerous signaling pathways, including corticotropin releasing hormone (CRH) and monoaminergic signaling which may contribute to the antidepressant actions of these compounds. Previous reviews have addressed this topic in depth [89], therefore, we will provide an overview of potential indirect mechanisms contributing to the antidepressant effects of NAS. Dysfunction of the hypothalamic-pituitary-adrenal (HPA) axis and excessive glucocorticoid and CRH signaling has been implicated in affective disorders, including depression. Allopregnanolone has been shown to normalize HPA axis function and restore homeostasis in CRH signaling (for review see [89]). The HPA axis is under tight GABAergic regulation which may underlie the ability of NAS to normalize neuroendocrine stress signaling. Similarly, the GABAergic system also regulates the monoaminergic system. The monoamine hypothesis of depression remains the prevailing theory of the underlying pathophysiology of depression. However, emerging evidence implicates the GABAergic system in contributing to monoamine deficits associated with depression and the antidepressant effects of SSRIs. SSRIs have been shown to increase the levels of NAS which has been proposed to contribute to the antidepressant effects of these compounds [90]. Recently, allopregnanolone has been demonstrated to influence dopamine release [91]. Given the crosstalk between GABAergic, CRF, and monoaminergic signaling, it is difficult to tease apart the exact mediators of the antidepressant actions of NAS.

Fluorescent NAS and photoaffinity labels have shown that NAS accumulate readily within cells, interacting with Golgi and perhaps other organelles in a structurally selective manner [92, 93]. One might hypothesize that these intracellular targets could contribute to beneficial effects. NAS targets may include proteins important for cellular stress responses, including autophagy and neuroinflammation [94–99]. Unnatural enantiomers of allopregnanolone and related NAS can engage intracellular pathways without substantially affecting ion channel targets [96, 100–104]. These tool compounds offer the possibility of parsing behavioral effects related to different molecular mechanisms.

Anti-inflammatory effects of NAS are of interest given hypothesized roles for inflammation in dysfunction in neuropsychiatric illness [105–108]. Allopregnanolone and progesterone exhibit anti-inflammatory effects in preclinical models, [109] and clinical studies have recently demonstrated a correlation between anti-inflammatory effect of brexanolone and clinical benefit [99]. These effects may occur via toll-like receptors-4 and -7 (TLR4/7), including binding the TLR adapter protein MyD88, but not TLR2 and TLR3 [97, 98, 110]. Pregnenolone promotes loss of TIRAP, an adapter protein for TLR2 and TLR4, which reduces secretion of inflammatory cytokines [110]. Microtubules are another possible locus for NAS anti-inflammatory effects. NAS photolabeling compounds bind the microtubule protein, β-tubulin, at cysteine-354, a site for colchicine, a compound with anti-inflammatory effects [93]. Further, NAS can alter the function of microtubules, and these effects contribute to antidepressant-like effects in rodents [111].

Some NAS can promote expression and trafficking of GABA_A_R subunits, including extrasynaptic receptor subunits affecting tonic inhibition. Effects may involve phosphorylation by protein kinase C (PKC) and other kinases [112]. Interestingly, increased receptor expression is triggered by allopregnanolone but not by the closely related synthetic NAS, ganaxolone [113]. Membrane progesterone receptors (mPRs) represent a class of G-protein-coupled receptors in the CNS that are activated by allopregnanolone and linked to phosphorylation of GABA_A_R subunits [114]. The NAS ORG OD 02-2 may selectively activate mPRs (without acute effects on GABA_A_Rs). ORG OD 02-2 increases GABA_A_R phosphorylation via cyclic AMP-dependent protein kinase (PKA) and PKC, resulting in increased GABA_A_R expression and enhanced tonic inhibition [115].

Allopregnanolone and related NAS also promote expression of nuclear receptors, including pregnane xenobiotic receptors (PXR) and liver xenobiotic receptors (LXR) [105, 116–119]. PXR/LXR may modulate steroid synthesis, among other pathways, to regulate cellular stress mechanisms [120], thereby contributing to anti-inflammatory and neuroprotective actions [119]. NAS interaction with PXR/LXR could also promote BDNF synthesis and signaling [121–123]. Downstream, the BDNF receptor TrkB can interact with antidepressant drugs through cholesterol [124]. These observations are of potential import because of the longstanding observations that BDNF and the plasticity induced by this neurotrophic factor have a role in the antidepressant effects of other treatments [125, 126].

Taken together, post-translational modification and protein expression changes represent attractive mechanisms to explain the durable, persisting effects on brain function observed in patients.

## NAS modulate emotional processing networks and behavioral states

Exogenous NAS have been shown to exert robust antidepressant and anxiolytic effects in both clinical and preclinical studies [22, 127, 128]. The role of neurosteroids in modulating affective states has been inferred from studies investigating the impact of neurosteroid deficits on mood. Levels of 5α-reductase, key rate limiting enzymes involved in neurosteroidogenesis, are reduced in the prefrontal cortex of individuals with major depressive disorder [129].

Cholesterol represents the upstream precursor of all neurosteroids, and the first step in neurosteroidogenesis is translocation of cholesterol from the plasma membrane to mitochondria by the actions of protein complexes that remain somewhat mysterious [130]. Both steroidogenic acute regulatory (StAR) proteins and translocator protein (TSPO), along with voltage-dependent anion channel (VDAC) interacting proteins, have been implicated. In the mitochondrion, P450 side-chain cleavage enzyme converts cholesterol to pregnenolone. The 3β-hydroxysteroid dehydrogenase enzyme converts pregnenolone to progesterone, which migrates across mitochondrial membranes to the cytoplasm, where 5α-reductase then 3α-hydroxysteroid dehydrogenase catalyze synthesis of allopregnanolone. As noted, 5α-reductase enzymes (of which there are three isoforms) are rate limiting so are a favored target for therapeutic development. Of experimental interest finasteride, dutasteride, and related compounds are 5α-reductase inhibitors. Ligands of TSPO are also enjoying interest as therapeutic agents in depression due to their potential impact on neurosteroidogenesis [131, 132].

One of the limitations impeding our knowledge regarding the role of endogenous neurosteroids is the difficulty in measuring endogenous neurosteroid levels and the inability to manipulate the enzymes involves in neurosteroid synthesis. Inhibition of 5α-reductase pharmacologically with finasteride induces behavioral deficits in animal models (for review see [128]). Human men treated with finasteride for male pattern baldness or benign prostatic hyperplasia can develop a condition termed post-finasteride syndrome. The syndrome is characterized by sexual dysfunction and negatively impacted mood [133], which is thought to involve impaired endogenous neurosteroidogenesis [134]. A recent study has demonstrated that knockdown of 5α-reductase type 1 and 2 in the basolateral amygdala (BLA) induces behavioral deficits in preclinical models with potential relevance to anxiety and depression [135]. These data suggest that endogenous 5α-reduced neurosteroids may play a role in setting a baseline affective tone.

As such, impaired endogenous neurosteroid synthesis has been implicated in the pathophysiology of both depression and anxiety (for review see [127]). A rapid decline in the endogenous levels of allopregnanolone during the peripartum period is thought to increase vulnerability to mood disorders during this period (for review see [136]). Thus, the mechanistic link to the established antidepressant effects of exogenous allopregnanolone analogs for the treatment of postpartum depression is clear. Treatment with exogenous allopregnanolone analogs is also showing robust antidepressant effects in major depressive disorder in which the mechanistic link is less clear. Recent preclinical studies have demonstrated that risk factors for psychiatric illnesses, such as chronic stress, alter the capacity for endogenous neurosteroid synthesis in preclinical models [128]. For example, social isolation (for review see [128]) and chronic unpredictable stress [135] decreased endogenous allopregnanolone levels and the levels of key neurosteroidogenic enzymes [137–140]. These data suggest that exogenous allopregnanolone treatment may exert its antidepressant effects potentially by supplementing deficits in allopregnanolone synthesis associated with the underlying neurobiology of disease.

As summarized above, the therapeutic efficacy of exogenous NAS treatment may be due to the evidence that deficits in endogenous neurosteroid signaling contributes to the underlying pathophysiology of mood disorders [141]. However, the mechanisms through which deficits in endogenous neurosteroidogenesis may contribute to mood disorders and the mechanisms mediating the antidepressant effects of exogenous NAS treatment remains unclear. Despite the fact that we have a good understanding of primary mechanisms of action of neurosteroids, how these mechanisms translate to antidepressant and anxiolytic effects is not clear.

**Fig. 3:**
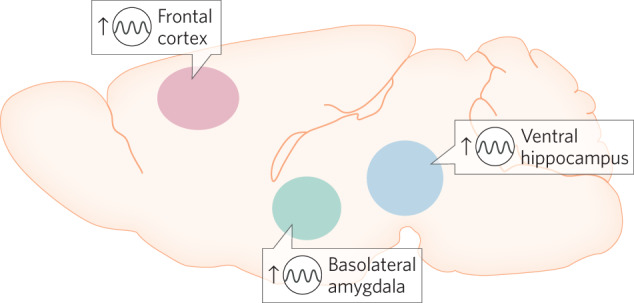
Circuit diagram. The diagram depicts oscillatory rhythms affected by NAS compounds in brain areas believed to be key to mood.

The persistent antidepressant effects observed in clinical trials with brexanolone and zuranolone treatment (see section “Clinical effectiveness of NAS for the treatment of depression”) suggest a mechanism of action other than actions as a GABA_A_R PAM. These sustained antidepressant effects may be mediated by the other known actions of NAS, such as the impact on autophagy, inflammation, other ion channels, or actions mediated by membrane progesterone receptors (see section “Possible non-GABA_A_R targets of relevance”). In addition, emerging evidence demonstrates that circuit network states drive behavioral states [142–145] and suggest that network-level changes play a role in the pathophysiology of psychiatric illnesses [146]. Psychiatric illnesses are increasingly acknowledged to involve network dysfunction [147]. In fact, many different approaches have demonstrated success in using EEG signatures as a diagnostic criterion for depression [148–151] as well as predict antidepressant response [152–155]. Commonalities observed in network-level changes associated with depression implicate specific brain regions contributing to the pathophysiology, including altered activity and connectivity within and between regions in the anterior cingulate cortex (ACC), dorsolateral prefrontal cortex, hippocampus, and amygdala (for review see [156]; Fig. 3). Similarly, studies in animal models demonstrate a unique role for the basolateral amygdala in mediating the impact of stress [157–159] and the anxiolytic and antidepressant effects of allopregnanolone [160–163]. These emerging studies point toward a role for NAS in modulating BLA network states to influence affective tone, such that deficits in NAS signaling in the BLA induces behavioral abnormalities and enhanced NAS signaling in the BLA improves behavioral outcomes. Consistent with this conceptual framework, preclinical studies have demonstrated altered network states (oscillations) associated with chronic stress-induced behavioral deficits [160] and the ability of NAS-based antidepressant treatments to alter network states across species [160, 164, 165] and improve behavioral outcomes following chronic stress [160]. It is important to note that the effects of NAS on network and behavioral states are unique from other GABA_A_R PAMs [160, 165, 166]. Recent evidence demonstrates that chronic stress reduces the capacity for endogenous neurosteroidogenesis [135] and that impaired endogenous neurosteroid signaling is sufficient to mimic the behavioral effects of chronic stress in mice [135]. This foundational preclinical work establishes a mechanism of action of the antidepressant effects of NAS-based treatments on network states and implicate impaired neurosteroidogenesis in the pathophysiological mechanisms underlying mood disorders.

## Future research directions

NAS represent a novel class of rapid-acting antidepressants with a different mechanism of action from previous antidepressant treatments. The robust preclinical and clinical evidence supporting the antidepressant effects of NAS suggest new mechanisms contributing to the underlying neurobiology of depression as well as offers the potential to identify new targets for the next generation of antidepressant treatments, including enantiomers, novel formulations, and targeting endogenous neurosteroid synthesis. This article tempers the enthusiasm for the potential of these rapid-acting antidepressant actions to revolutionize treatment options for patients with a discussion of potential limitations for consideration. The mechanisms whereby NAS exert antidepressant effects are not fully understood but emerging evidence suggests the ability of these compounds to alter network states involved in emotional processing to influence affective states. Finally, the ability of NAS to modulate network and behavioral states relevant to depression is unique to this class of compounds and is unique from other GABA agonists, such as benzodiazepines. These emerging findings elucidate the antidepressant mechanisms of these compounds as well as provide information regarding the underlying neurobiology of disease.
